# ADP heptose, a novel pathogen-associated molecular pattern identified in *Helicobacter pylori*

**DOI:** 10.1096/fj.201802555R

**Published:** 2019-06-21

**Authors:** Lennart Pfannkuch, Robert Hurwitz, Jan Traulsen, Janine Sigulla, Marcella Poeschke, Laura Matzner, Paul Kosma, Monika Schmid, Thomas F. Meyer

**Affiliations:** * Department of Molecular Biology, Max Planck Institute for Infection Biology, Berlin, Germany;; † Department of Infectious Diseases and Pulmonary Medicine, Charité, University Hospital Berlin, Berlin, Germany;; ‡ Berlin Institute of Health, Berlin, Germany;; § Department of Chemistry, University of Natural Resources and Life Sciences–Vienna, Vienna, Austria

**Keywords:** NF-κB, ALPK1, TIFA, LPS, PAMP

## Abstract

The gastric pathogen *Helicobacter pylori* activates the NF-κB pathway in human epithelial cells *via* the recently discovered α-kinase 1 TRAF-interacting protein with forkhead-associated domain (TIFA) axis. We and others showed that this pathway can be triggered by heptose 1,7-bisphosphate (HBP), an LPS intermediate produced in gram-negative bacteria that represents a new pathogen-associated molecular pattern (PAMP). Here, we report that our attempts to identify HBP in lysates of *H. pylori* revealed surprisingly low amounts, failing to explain NF-κB activation. Instead, we identified ADP-*glycero*-β-D-*manno*-heptose (ADP heptose), a derivative of HBP, as the predominant PAMP in lysates of *H. pylori* and other gram-negative bacteria. ADP heptose exhibits significantly higher activity than HBP, and cells specifically sensed the presence of the β-form, even when the compound was added extracellularly. The data lead us to conclude that ADP heptose not only constitutes the key PAMP responsible for *H. pylori*–induced NF-κB activation in epithelial cells, but it acts as a general gram-negative bacterial PAMP.—Pfannkuch, L., Hurwitz, R., Traulsen, J., Sigulla, J., Poeschke, M., Matzner, L., Kosma, P., Schmid, M., Meyer, T. F. ADP heptose, a novel pathogen-associated molecular pattern identified in *Helicobacter pylori*.

The innate immune system provides mammalian cells with various means of self-defense against microbial infections. A key element in detecting possible threats is the recognition of pathogen-associated molecular patterns (PAMPs) by host cells (1). These highly conserved structures are found in bacteria, viruses, and fungi and can be detected *via* pattern-recognition receptors (2). Although a variety of PAMPs has been identified in the last decades (*e*.*g*., LPS, CpG nucleotides, and flagellin) the picture is still incomplete (3). Recognition of these structures alerts the host cell to the presence of potentially harmful microorganisms and triggers innate immune signaling pathways that induce the production of inflammatory mediators and attract immune cells to fight an infection.

One of the main pathways activated in response to infection with pathogenic bacteria is NF-κB signaling, which has been implicated in inflammation and carcinogenesis (4). Canonical NF-κB signaling can be stimulated by a variety of effectors, such as microbial PAMPs or pro-inflammatory cytokines like TNF-α (5). Activation of the upstream receptors leads to phosphorylation of the inhibitor of IKK-β and subsequent phosphorylation of the inhibitor of NF-κB-α (IκB-α). This induces polyubiquitination and proteasomal degradation of IκB-α, which releases the p65/p50 heterodimer normally sequestered in the cytoplasm. Upon release, p65/p50 is translocated into the nucleus, where it activates the expression of multiple genes involved in inflammation and proliferation (6).

LPS is a well-studied PAMP found in most gram-negative bacteria. The inner core of LPS typically consists of 3-deoxy-d*-manno-*octulosonic acid and l,d-heptose units (7). The precursor of the l,d-heptose units, ADP-l-*glycero*-β-d-*manno*-heptose (ADP heptose), is synthesized in a 5-step pathway, starting from d-sedoheptulose 7-phosphate, which is *1*) converted into d-*glycero*-α, β-d-*manno*-heptose-7-phosphate by the sedoheptulose-7-phosphate isomerase GmhA, *2*) phosphorylated to form d-*glycero*-β-d-*manno*-heptose-1,7-bisphosphate (HBP) by the bifunctional kinase or adenylyltransferase HldE, *3*) dephosphorylated to d-*glycero*-β-d-*manno*-heptose-1-phosphate by the d-*glycero*-β-d-*manno*-heptose-1,7-bisphosphate-7-phosphatase (GmhB), followed by *4*) nucleotide activation by formation of ADP-d-*glycero*-β-d-*manno*-heptose by HldE, and, finally, *5*) epimerization to ADP heptose by the epimerase HldD (7) (Supplemental Fig. S1*A*). The resulting activated heptose unit is then integrated into the LPS core region.

In 2015, the intermediate metabolite HBP was identified as a potent effector of NF-κB activation by *Neisseria *species, which is released into the cytosol of infected epithelial cells *via* phagocytosis followed by lysosomal degradation (8). More recently, we showed in gastric epithelial cells infected with *Helicobacter pylori* that the proteins α-kinase 1 (ALPK1) and TRAF-interacting protein with forkhead-associated domain (TIFA) are key components in the response to HBP, leading to activation of canonical NF-κB signaling (9). In this system, too, NF-κB activation was stimulated by HBP upon transfection as well as, presumably, upon translocation to the cytosol *via* the type-IV secretion system (T4SS) (9).

The discovery of ALPK1 as an NF-κB pathway–associated signaling molecule (9, 10) and a novel pattern-recognition receptor acting *via* the rapid formation of TIFAsomes, a previously unknown signaling platform, represent major breakthroughs in the field. However, even in our original study, we were unable to isolate HBP from *H. pylori* for use in experiments and instead had to rely on chemically synthesized HBP. Since then, we have made an intense effort to confirm the presence of HBP in bacterial lysates using a mass spectrometry (MS) approach. Chemical derivatization of β-HBP finally allowed us to overcome the detection limit and revealed that HBP was present at concentrations far lower than those required to activate NF-κB with synthetic HBP. We thus hypothesized that yet another compound involved in LPS synthesis might be responsible. After screening fractionated *H. pylori* lysates for their capacity to stimulate NF-κB, we identified ADP heptose by MS as a novel, potent NF-κB–activating PAMP acting *via* the ALPK1-TIFA signaling axis [see also Pfannkuch *et al.* (11)]. In parallel to our studies, Zhou *et al.* (12) recently demonstrated that ADP heptose is indeed the ligand for ALPK1. Because ADP heptose is present in most LPS-containing bacteria, this molecule likely is of relevance for numerous infections with gram-negative bacteria.

## MATERIALS AND METHODS

### Cell culture

AGS cells stably expressing a tdTomato-TIFA-Flag construct (tdTomato construct from Takara, Kyoto, Japan) were created using the pMW1064 (td-Tomato/TIFA-Flag/Kan) plasmid. The tdTomato-TIFA-Flag gene was shuttled in a lentiviral expression plasmid (pLenti ×1 Zeo Dest, Plasmid 17299; Addgene, Watertown, MA, USA). Cells were lentivirally transduced and selected with zeocin, and a cell line was created from single clones. AGS cells stably expressing an NF-κB-luc2P construct were created using the pGL4.32 (luc2P/NF-κB-RE/Hygro) plasmid (Promega, Madison, WI, USA). The NF-κB response element and the luc2P reporter gene was shuttled in a pLenti plasmid (pLenti phosphoglycerate kinase Neo Dest, Plasmid 19067; Addgene). Cells were lentivirally transduced and selected with neomycin, and a cell culture was created from single clones. Clustered regularly interspaced short palindromic repeat (CRISPR)/CRISPR-associated protein 9 (Cas9) control and ALPK1 or TIFA knockout cells were created as previously described by Zimmermann *et al*. (9) from the AGS parent cell line [1739; American Type Culture Collection (ATCC), Manassas, VA, USA]. All cells were cultivated at 37°C and 5% CO_2_ in Roswell Park Memorial Institute medium (Thermo Fisher Scientific, Waltham, MA) with 10% heat-inactivated fetal calf serum (9).

### Bacterial culture and lysis preparation

*H. pylori* wild-type (WT) strain P12 and corresponding mutants were grown on GC agar plates supplemented with 10% horse serum and vancomycin or respective antibiotic (**Table 1**) and cultivated for 2 passages at 37°C and 5% CO_2_ (13). All other strains (Table 1) were grown on standard Luria-Bertani (LB) medium agar plates and cultivated for 2 passages at 37°C (or 26° in case of *Yersinia* *enterocolitica*) and 5% CO_2_.

**TABLE 1:** Bacterial Strains and Cultivation

Strain	Name	Plate
X 52	*P. aeruginosa*	LB-Agar
X 98	*S. typhimurium*	LB-Agar
X 2	*S. pneumoniae*	LB-Agar
X 466	*Y. enterocolitica*	LB-Agar but 26°C
X 122	*L. monocytogenes*	LB-Agar
P511	*H. pylori P12 WT*	GC agar + vancomycin
P587	*H. P12*Δ*rfaE*	GC agar + vancomycin + kanamycin
P589	*H. pylori P12*Δ*gmhB*	GC agar + vancomycin + kanamycin
P587	*Helicobacter pylori P12*Δ*gmhA*	GC agar + vancomycin + kanamycin
P582	*Helicobacter pylori P12*Δ*rfaD*	GC agar + vancomycin + kanamycin

For lysate preparation, bacteria were harvested by resuspension in water and diluted to optical density at 550 nm (OD_550_) 1. Cells were lysed by heating to 95°C for 15 min. Lysates were centrifuged at 4000 *g* for 3 min and supernatants filtered through a 0.22-µm syringe filter. To validate lysis, 100 µl of lysate were incubated on LB medium agar plates overnight at 37°C or 26°C at 5% CO_2_ (Table 1).

### Carbohydrate delivery by lipofection and luciferase assay

AGS cells stably overexpressing an NF-κB-luc2P construct were starved in serum-free medium for 2 h at 37°C and 5% CO_2_ prior to sugar delivery. Delivery of sugar into the cytoplasm was achieved by treating cells at 70% confluence with a preparation of 1 µl sugar solution diluted to respective concentrations mixed with 7.5 µl Opti-MEM (Thermo Fisher Scientific), 6.25 µl ATP (20 mM; Roche, Basel, Switzerland), and 1.25 µl lipofectamine 2000 (Thermo Fisher Scientific) after 20 min preincubation at room temperature. Alternatively, sugars were added to the starvation medium without transfection reagent. Cells were incubated for 3 h at 37°C and 5% CO_2_, and the subsequent lysis and luciferase assay was performed according to the manufacturer’s instructions (Promega).

### Confocal microscopy

Stably tdTomato-TIFA–overexpressing cells were seeded on glass cover slips and grown for 24 h at 37°C and 5% CO_2_. Cells were either transfected with β-d-ADP-heptose or β-HBP using lipofectamine 2000 (Thermo Fisher Scientific) under serum-free conditions, treated with sugars without transfection, or left untreated for 3 h at 37°C and 5% CO_2_. To visualize TIFAsome formation, cells were fixed with 4% (w/v) paraformaldehyde and counterstained with DAPI (1:300, H1840-10; Roche) for 2 h. Cover slips were mounted with Mowiol 40–88 (324590; MilliporeSigma, Burlington, MA) and analyzed by laser scanning microscopy using a Leica SP8 confocal microscope (Leica Microsystems, Buffalo Grove, IL, USA) (9). For quantification of TIFAsome-positive cells, 2 images with a ×40 magnification were obtained for every condition. Dead or dividing cells (as judged by a condensed, irregular nucleus) were excluded from analysis. Nuclei and TIFAsome-positive cells were counted using the Counter plugin of Fiji (*https://fiji.sc/*).

### Quantitative RT-PCR

Quantitative RT-PCR (qRT-PCR) was performed using the Power Sybr Green RNA-to-CT 1-Step Kit according to the manufacturer’s instructions (Thermo Fisher Scientific). As previously described by Zimmermann *et al*. (9), 1 ng/µl of the isolated RNA was added per reaction, and the final concentration of the primers was 166 nM. qRT-PCR was performed using a StepOnePlus Real-Time PCR system (Thermo Fisher Scientific) with a 1-step protocol for both RT reaction and PCR reaction. The following conditions were used during the protocol: an initial cycle for the generation of cDNA at 48°C for 30 min; second cycle for the activation of the hot-start Taq polymerase at 95°C for 10 min; and 40 consecutive denaturation cycles at 95°C for 15 s, each followed by primer annealing at 60°C for 1 min. Baseline and *C_t_* values were determined automatically by the StepOne Software v.2.3 (Thermo Fisher Scientific). Fold changes were calculated using the 2^−ΔΔ^*^Ct^* method (14). The following primers were used: IL-8 5′-ACACTGCGCCAACACAGAAAT-3′ and 5′-ATTGCATCTGGCAACCCTACA-3′, GAPDH 5′-GGTATCGTGGAAGGACTCATGAC-3′ and 5′-ATGCCAGTGAGCTTCCCGTTCAG-3′.

### *Glycero*-d-*manno*-heptoses

d-*glycero*-d-*manno-*HBP (both α- and β- configurations), d-*glycero*-d-*manno*-heptose 7-phosphate (H7P), β-d- and β-l-*glycero*-d-*manno*-heptose-1-phosphate (β-H1P) were chemically synthesized as previously described (15–17).

### ADP heptose

ADP heptoses were synthesized chemoenzymatically with H1P or H7P as glycosyl donors, respectively, or chemically with β-H1P or with peracetylated l-*glycero*-d-*manno*-heptose. The following strategies were applied: *1*) We coupled the unprotected l- and d-*glycero*-d-*manno*-heptose-1-phosphate to adenosine-5′-phosphomorpholidate (AMP morpholidate) under anhydrous conditions in pyridine or tetrazole as described by Zamyatina *et al.* (17) and Wagner *et al.* (18). *2*) We coupled β-H1P (d and l) to AMP with 2-chloro-1,3-dichloromethylimidazolinium chloride in D_2_O as described by Tanaka *et al* (19). Both the AMP morpholidate and the dichloromethylimidazolinium chloride or imidazole methods were carried out with up to 100 nmol H1P as starting material, keeping the reaction volume as small as possible (1-5 µl). After completion of the reaction, the mixtures were diluted with 50 µl water, and ADP heptoses were extracted with 2 vol. of chloroform and methanol (2:1). The aqueous phase was dried and the residue dissolved in 50 µl water and separated on a reversed-phase HSS T3 ultraperformance liquid chromatography (UPLC) column. Products were also analyzed by liquid chromatography (LC) electrospray ionization (ESI) MS as described below. *3*) β-l-ADP heptose was synthesized according to the protocol previously described by Zamyatina *et al.* (17) but with a modification regarding the introduction of the phosphate: 50 mg of the peracetylated l-*glycero*-d-*manno*-heptose were deacetylated with Hünig’s base as described. The resulting 2,3,4,6,7-penta-*O*-acetyl-*manno*-heptopyranose was purified by silica chromatography and phosphitylated with bis(2-cyanoethoxy)-*N*,*N*-diisopropylaminophosphine in dry acetonitrile/tetrazole in the presence of 1-hydroxy-6-(trifluoromethyl)benzotriazole (20). The phosphitylating reagent was prepared by stirring 60 mg 1-hydroxy-6-(trifluoromethyl)benzotriazole with 1 ml 0.1 cM bis(2-cyanoethoxy)-*N*,*N*-diisopropylaminophosphine in anhydrous acetonitrile for 2 h under reduced pressure. The mixture was slowly added to 10 mg of the penta-*O*-acetyl heptopyranose in 1 ml anhydrous acetonitrile containing 3% tetrazole over 30 min, and the reaction was incubated for 8 h at ambient temperature. Oxidation to the phosphate ester was carried out by slowly adding 50 µl of 10% tert-butylhydroperoxide over 1 h on ice. The reaction mixture was diluted with 5 vol. dichloromethane and washed with brine and 0.5 M triethylammonium bicarbonate (TEAB; pH 8.5), successively. The organic phase was dried under nitrogen, dissolved in 500 µl acetonitrile, and passed over a silica gel 60 column, which was eluted with chloroform and methanol and water (3:2:0.1), yielding 2 components with the expected molecular mass for the penta-*O*-acetyl-heptose-1-(bis-cyanoethyl)-phosphate of *m/z* 629.2 [M+Na]^+^ (ESI-MS). The later eluting sugar phosphate (assumed to be the β-anomer) was dried and dissolved (5 mg) in 500 µl dry pyridine. Cyanoethyl groups were cleaved by adding 200 µl bis(trimethylsilyl)acetamide and 10 µl 1,8-diazabicyclo[5.4.0]undec-7-ene. The reaction was stirred for 20 min at ambient temperature (21), then stopped by diluting with 5 vol dichloromethane and methanol (2:1) and 2 vol 500 mM TEAB (pH 8.2). The organic layer was dried, solubilized in 50% methanol, and separated on an HSS T3 high-performance LC column that was isocratically eluted with 40% acetonitrile in 10 mM TEAB. Heptose-phosphate fractions were detected by ESI-MS (Waters QDa; Waters, Milford, MA, USA), dissolved in anhydrous dichloromethane, dried under vacuum over phosphorpentoxide, and dissolved in anhydrous pyridine. Coupling to AMP morpholidate and deprotection was performed essentially as described. The resulting *O*-acetylated-ADP heptose was deprotected with 50 mM triethylamine (pH 10.5) in methanol or TEAB. The product was further purified by anion exchange chromatography (Supelcosil LC-SAX 25 cm × 4.6 mm; 5-µm particle). ADP heptose was eluted with a linear gradient of 10–500 mM TEAB in water [total yield: 0.65 mg (1.3%); ESI-MS (negative mode): *m/z* 618.05]. *4*) l/d-α-ADP heptose was synthesized as previously described by Zamyatina *et al.* (17).

### Enzymatic synthesis of β-ADP heptose from β-H1P or H7P

Up to 200 nmol d-β-H1P (triethylammonium salt) was incubated at 35°C with 1 µg recombinant HldE from *H. pylori* in 20 µl HldE buffer [50 mM Tris HCl (pH 8.0), 150 mM NaCl, 5 mM MgCl_2_, and 20 mM ATP] for 1 h. The reaction was stopped by addition of 50 µl chloroform and methanol (2:1). The aqueous phase was dried in a SpeedVac (Thermo Fisher Scientific) evaporator and dissolved in 50 µl water. The product was analyzed by LC-MS as described below. When H7P was used instead of H1P, the above reaction was performed in the presence of 1 µg of recombinant GmhB from *H. pylori*.

### Enzymatic synthesis of β-HBP from H7P

d-H7P (10 nmol, triethyl ammonium salt) was incubated with 1 µg recombinant RfaE from *Neisseria gonorrheae* in 20 µl HldE buffer [50 mM Tris HCl (pH 8.0), 150 mM NaCl, 5 mM MgCl_2,_ and 20 mM ATP] for 1–8 h at 35°C. To destroy minor amounts of ADP heptose, which were formed because of the presence of the endogenous *Escherichia coli* adenylyltransferase activity, 1 µl phosphodiesterase (PDE) (*Crotalus adamanteus*; 3 mU/ml) was added for 30 min. The reaction was stopped by addition of 50 µl chloroform and methanol (2:1). The aqueous phase was dried in a SpeedVac evaporator and dissolved in 50 µl water. The product was analyzed by LC-MS as described below.

### Derivatization of heptoses with 3-amino-9-ethylcarbazole

Sugar-phosphates were dephosphorylated with calf intestine alkaline phosphatase (CIP) or by acid hydrolysis with trifluoroacetic acid (TFA) (50 mM, 30 min, 90°C). Reactions were conducted in 10 µl. After hydrolysis, samples were diluted with 50 µl methanol and dried in a SpeedVac evaporator. For reductive amination, the hydrolyzed samples were incubated with 100 µl 3-amino-9-ethylcarbazole (AEC) (60 mM in methanol) followed by the addition of 25 µl of sodium cyanoborohydride (50 mM in water) and 20 µl of acetic acid. After 1 h at 60°C, samples were directly submitted to UPLC analysis. (BEH C18, 50 × 2.1 mm; Waters). Compounds were eluted with a linear gradient of 10–100% acetonitrile in 0.1% formic acid.

### Matrix-assisted laser desorption/ionization time-of-flight tandem MS

Lyophilized samples were solubilized with 4 µl 2% acetonitrile and 0.1% TFA. One microliter of each sample was mixed with an equal volume α-cyano-4-hydroxycinnamic acid (0.5% solubilized in 60% acetonitrile and 0.3% TFA) and transferred onto the matrix-assisted laser desorption/ionization (MALDI)/MS template. MS and tandem MS (MSMS) spectra for AEC sugars were acquired in positive mode with a 4700 Proteomics Analyzer (Sciex, Framingham, MA, USA) MALDI time-of-flight (TOF)/TOF instrument. MS mass range was set to 300–600 Da. ADP heptose measurements were acquired in negative mode, and the MS mass range was set to 250–1000 Da. All data were evaluated manually.

### UPLC

Carbohydrates were separated by ion-pairing reversed-phase chromatography on an HSS T3 UPLC column (2.1 × 150 mm, 1.8 µm; Waters) with a linear gradient of 10 mM TEAB, (pH 8.5) from 2% acetonitrile to 90% acetonitrile (containing 10 mM TEAB) over 7 min at 45°C and a flow rate of 0.4 ml/min. Eluted compounds were detected by UV absorbance (photodiode array detector; Waters) and by ESI-MS detection (QDa; Waters) or MALDI-TOF MSMS.

The QDa was operated in an electrospray negative ion mode by applying a voltage of 0.8 kV. The cone voltage was set at 15 V. The probe temperature was set at 600°C. A full mass spectrum between *m*/*z* 100 and 1200 was acquired at a sampling rate of 8.0 points/s.

For quantification of ADP heptose, calibration standard solutions at 7 concentrations from 10 to 100 pmol/µl were analyzed in single ion recording (SIR) mode at *m/z* 618. The calibration curves were established by plotting the peak areas (*m/z* 618) against the concentrations of analytes with linear regression analysis.

### Cloning and expression of the ADP heptose pathway enzymes RfaE, HldE, and GmhB in *E. coli*

DNA recombinant procedures were performed according to standard methods described by Sambrook *et al.* (22). DNA encoding the enzymes of the ADP heptose pathway were cloned from *H. pylori* P12 (GmhB), *H. pylori* G27 (HldE), and Neisseria *meningitides* 2C43 (RfaE). DNA templates were PCR amplified with the primers indicated below. The PCR products were digested with the indicated restriction enzymes and ligated to plasmid vector pGex-2T prelinearized with the same restriction enzymes, yielding pGex-2T-RfaE, pGex-2T-HldE, and pGex-2T-GmhB.

*E. coli* strain BL21(DE3) was used and maintained at 37°C using LB medium. For strain activation, 0.5 ml of an overnight culture were inoculated into 50 ml LB medium and grown at 37°C to OD_600_ 0.5. To express the glutathione S-transferase–tagged enzymes, the 3 constructed recombinant plasmids (pGex-2T-RfaE, pGex-2T-HldE, and pGex-2T-GmhB) were transformed into *E. coli* strain BL21(DE3) to allow evaluation of protein expression. The recombinant strains were grown in LB medium in the presence of ampicillin (50 μg/ml) at 37°C.

### Primers

The following primers were used: RfaE kinase: RfaE_fw_*Bam*HI: 5′-GACTGGATCCTCCGCCAAGTTCCAACAAG-3′ (fw, forward), RfaE_rv_*Eco*RI: 5′-CCCGGAATTCCTACATTGTTGATTGCCCTGAC-3′ (rv, reverse); HldE bifunctional kinase and adenylyl transferase: HldE_fw_*Bam*HI: 5′-GACTGGATCCATGAAAAAAATCTTAGTCATAGGC-3′, HldE_rv_*Eco*RI: 5′-CCCGGAATTCCTTCAATCATTGCATGTCC-3′; GmhB phosphatase: GmhB_fw_*Bam*HI: 5′-GACTGGATCCATGAACACTAACAAAGCCC-3′, GmhB_rv_*Eco*RI: 5′-CCCGGAATTCCTTTATTTGATTAGATCTATCATCTCTTTAA-3′.

The templates were as follows: for RfaE (AAF41238), *N. meningitides* 2C43 (strain N1072); for HldE (O255529), *H. pylori* G27 (strain P224); for GmhB (D64627), *H. pylori* P12 (strain P511).

### Purification of enzymes

Proteins were expressed in 1-L cultures, respectively. The following expression and purification protocol was applied per 100 ml of bacterial culture: 5 ml preculture was grown for 16 h at 37°C, followed by the addition of 95 ml of prewarmed LB medium with ampicillin. At the OD_600_ of 1.5, protein expression was induced for 4 h at 30°C with 1 mM isopropyl β-d-1-thiogalactopyranoside. Cells were harvested by centrifugation (4000 *g*, 4°C, 15 min). The cell pellet was frozen in liquid nitrogen, thawed on ice for 15 min, and resuspended in 3 ml of lysis buffer [20 mM Tris (pH 8.0) and 5 mM EDTA]. Fifty microliters of lysozyme solution (10 mg/ml) was added and incubated on ice for 30 min. One microliter of benzonase grade II (1 U/µl; Merck, Kenilworth, NJ, USA) and 50 µl of 1 M MgCl_2_ was added and incubated on ice for 30 min. The lysates were centrifuged at 6000 *g* at 4°C for 30 min. The supernatants were mixed with 200 µl of glutathione-sepharose 4B beads (50% suspension in PBS) for 4 h and washed several times with PBS. Bound proteins were eluted with 20 mM glutathione in 100 mM Tris HCl (pH 8.0). Eluates were applied on a sephadex G20 column equilibrated in PBS to get rid of glutathione. Protein concentration was adjusted to 10 mg/ml by ultrafiltration centrifugal devices and stored in aliquots at −80°C.

### Isolation of ADP heptose from *H. pylori*

*H. pylori* strain P12 was grown on horse serum agar plates supplemented with vancomycin (10 µg/ml) and cultivated for 2 passages at 37°C and 5% CO_2_ (13). For lysate preparation, bacteria were harvested by resuspension in water and diluted to OD_550_ 1. Cells were lysed by heating to 95°C for 15 min. Lysates were centrifuged at 4000 *g* for 3 min and supernatants filtered through a 0.22-µm syringe filter.

1 ml of the cleared lysate was extracted by addition of 2 ml chloroform/methanol (2:1). After vortexing and centrifugation (5 min, 10,000 *g*), the aqueous phase was passed over a HyperSep solid phase extraction (SPE) aminopropyl cartridge (200 mg/3 ml), which was equilibrated with 50 mM acetic acid in 50% methanol. Bound compounds were eluted with 500 mM TEAB (pH 8.5) in 50% methanol. Eluates were dried in a SpeedVac evaporator, solubilized in 1 ml 10 mM ammonium bicarbonate (pH 8.0), and passed over a graphite carbon column [Supelclean Envi-Carb (MilliporeSigma), 1 ml]. Sugars were eluted with 30% acetonitrile. The eluate was dried in a SpeedVac evaporator, dissolved in 10 µl 10 mM TEAB buffer, and loaded onto an HSS T3 reversed-phase UPLC column (3 × 150 mm, 1.8 µm) equipped with an HSS T3 VanGuard precolumn. Compounds were separated at a flow rate of 0.4 ml/min at 45°C with a linear gradient from 2% acetonitrile to 80% acetonitrile containing 10 mM TEAB as an ion-pairing reagent. Fractions were analyzed by UV detection.

### Deletion of *rfaE*, *hldD*, and *hldA* genes in *H. pylori*

Deletion of *rfaE*, *hldD,* and *hldA* was performed as previously described (9, 23). The up- and downstream regions (0.5 kb) of the respective genes were amplified, gel-purified, digested using *Bam*HI, and ligated. After ligation, a column purification was performed and the fragments amplified by PCR. The 1-kb products of up- and downstream fragments were cloned into pGEM-T Easy (Promega) and separated by the kanamycin resistance gene at the *Bam*HI restriction site. The plasmid was then transformed into dam-negative *E. coli* strain GM2199. After isolation, plasmid DNA was used to transform *H. pylori* P12 WT (P511). *H. pylori* was selected using kanamycin-containing agar plates, and gene knockout was confirmed by PCR amplification using primers spanning DNA up- and downstream of the sites.

### Western blot

Infected cells were lysed in 2× Laemmli buffer with incubation at 96°C for 5 min, separated by 10% SDS-PAGE, transferred to PVDF membranes, and blocked in Tris-buffered saline buffer supplemented with 0.05% Tween 20 and 5% nonfat milk for 30 min. Samples were probed against primary antibodies at 4°C overnight. After washing with Tris-buffered saline buffer supplemented with 0.05% Tween 20, membranes were probed with matching secondary horseradish peroxidase–conjugated antibodies (1:3000; Amersham, Little Chalfont, United Kingdom) and visualized with enhanced chemiluminescence reagent (PerkinElmer, Waltham, MA, USA). The following primary antibodies were used: anti-phosphotyrosine 99 (pY99) sc-7020, anti–cytoxin-associated gene A (b300) sc-25766, and β-actin A5441 from MilliporeSigma.

### Statistical analysis

Graphs were prepared using GraphPad Prism v.7 software (GraphPad Software, La Jolla, CA, USA) or Microsoft Excel. Data are presented as means ± sem. Two-sided unpaired Student’s *t* test was used to calculate *P* values. Significance was assumed if *P* < 0.05. The number of replicates is provided in the figure legends.

## RESULTS

### *H. pylori* lysates contain only minor amounts of HBP

In order to confirm the presence of HBP in *H. pylori* lysates, we tried to separate the heptose by SPE methods based on the protocol described by Gaudet *et al.* (8). Detection of HBP was performed by ion-pairing reversed-phase chromatography *via* ESI-MS. However, in SPE extracts derived from at least 1 ml of *H. pylori* lysate, we could not detect HBP as judged by the absence of a mass with *m/z* 369 [M-H]^−^ (unpublished results). Because the detection limit of HBP was relatively high (≥100 pmol), we aimed to increase the sensitivity by derivatizing the heptose by reductive amination using AEC after treatment with either CIP, which cleaves both the 7′-*O*-phosphate and the glycosidic phosphate ester, or with TFA, a mild acid that hydrolyzes only the glycosidic binding (24). This creates an aldehyde group enabling Schiff’s base generation between carbohydrates and primary amines, a reaction that does not occur if the sugar is esterified to phosphate by an *O-*glycosidic bond (Supplemental Fig. S1*B*). We then determined the sensitivity of our assay using chemically synthesized β-HBP as a reference.

**Figure 1:**
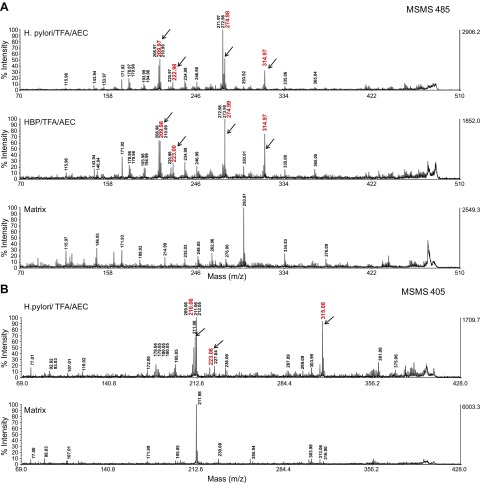
Detection of AEC-derivatized heptoses in *H. pylor*i extracts by MALDI-TOF MSMS. *A*) HBP or extracts from *H. pylori* (graphite carbon eluate) were derivatized with AEC after acid hydrolysis, respectively. AEC heptose adducts were separated with UPLC and analyzed by MALDI-TOF MSMS. Shown are the MSMS spectra of the precursor ion *m/z* 485 [M+H]^+^ as the putative Schiff base–derived H7P-AEC adduct. Upper panel: Derivatization of *H. pylori* extracts, arrows and numbers in red: fragment ions (*m/z* 210, 223, 275 and 315) of the precursor ion *m/z* 485 [M+H]; middle: derivatization of HBP, lower: matrix control. *B*) Upper panel: MALDI-MSMS spectrum of the AEC-*glycero*-d-*manno*-heptose precursor ion (*m/z* 405 [M+H]^+^) derived from reductive amination of an *H. pylori* extract after acid hydrolysis with TFA; arrows and numbers in red: fragment ions (*m/z* 210, 223, 315) of the precursor ion *m/z* 405 [M+H]. Lower: matrix control.

Derivatization of β-HBP allowed us to detect AEC sugar derivatives by MALDI-TOF MSMS with a detection limit of 1 pmol. Thus, we detected precursor masses of *m/z* 405 [M+H]^+^ for the CIP-treated HBP, with both phosphates cleaved and *m/z* 485 [M+H]^+^ after TFA hydrolysis, removing only the *O-*glycosidically bound phosphate. Reductive amination of the *H. pylori* SPE extract after acid hydrolysis led to the appearance of the predicted *m/z* 485 mass and fragment ions (*m/z* 210, 223, 275, and 315) (**Fig. 1*A*** and Supplemental Fig. S1*C*), but it also revealed that HBP was present in quantities too low to account for the stimulation of NF-κB. In our experiments with synthetic HBP, a concentration of at least 10 µM was required (9). By contrast, in extracts (containing 9 × 10^8^ bacteria/ml), only concentrations of <0.1 µM HBP could be detected. To our surprise, in the lysates we also detected a mass of *m/z* 405 (fragment ions of *m/z* 210, 223, and 315) after AEC derivatization, which appeared after acid treatment (Fig. 1*B* and Supplemental Fig. S1*C*), indicative of a heptose with an acid labile *O*-glycosidic bond.

**Figure 2:**
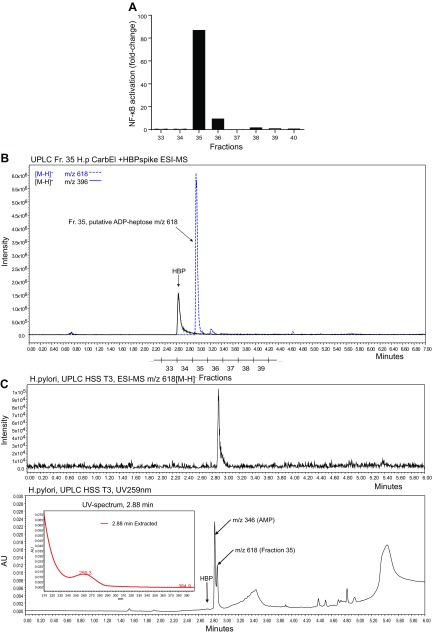
Identification of a novel NF-κB–stimulating compound in *H. pylori* lysates. *A*) AGS NF-κB luciferase reporter cells were incubated with *H. pylori* fractions separated by ion-pairing reversed-phase UPLC. Fractions were mixed with transfection reagent (lipofectamine 2000) and then added to NF-κB luciferase reporter cells for 3 h. Induction of NF-κB was determined by measurement of luciferase activity. Treatment with lipofectamine 2000 was used as negative control. Induction is shown as fold change compared with a negative control. Representative result of 2 repeated runs is shown. *B*) *H. pylori* extract was spiked with β-HBP. Shown are the chromatograms in SIR mode for *m/z* 369 (black line) and *m/z* 618 (negative mode) (blue, dashed line). Corresponding fractions in *A* are given below. *C*) *H. pylori* extract (carbon graphite eluate) was separated by UPLC. Shown are the chromatograms in SIR mode for *m/z* 618 (upper panel) and the UV absorbance at 259 nm (lower panel).

In order to identify the compounds present in *H. pylori* that could stimulate NF-κB activation, we fractionated bacterial lysates by UPLC. Improved chromatographic resolution of the *H. pylori* carbohydrate compounds from the SPE extract was achieved by ion-pairing reversed-phase UPLC. We tested the fractions for their ability to stimulate NF-κB in luciferase reporter cells and for the appearance of AEC-derivatized heptoses using MALDI MS. By transfecting the fractions into reporter cells, we found that only a single fraction in any given run had a strong capacity for inducing NF-κB (**Fig. 2*A***). For each of these NF-κB–stimulating fractions, the most prominent MSMS peak intensities (*m/z* 223) of the AEC precursor masses were quantified (summarized in Supplemental Table S1; see also Supplemental Figs. S2*A*, *B* and S3*A*, *B*). An AEC sugar of *m/z* 405 [M+H]^+^ was detected only after acid treatment but not after digestion with CIP (Supplemental Fig. S1*D*). From these data, we concluded that not HBP but rather a *manno*-heptose with an acid labile glycosidic linkage partly resistant to alkaline phosphatase was present in the NF-κB–stimulating fractions. This was further supported by the observation that the active fraction contained a prominent compound with a mass of *m/z* 618 [M-H]^−^ (ESI-MS) and a UV absorbance of 259 nm, whereas spiked HBP eluted earlier (Fig. 2*B, C*). We therefore hypothesized that the substance could be β-ADP heptose, the final metabolite of the ADP heptose pathway (Supplemental Fig. S1*E*). This assumption was further supported by the fact that treatment of the active *H. pylori* fraction after UPLC chromatography with alkaline phosphatase (CIP) or with PDE from *C. adamanteus* showed that the active compound was completely hydrolyzed by PDE. By contrast, CIP treatment caused only minor hydrolysis after prolonged digestion times (Supplemental Fig. S3*C*).

### *De novo* synthesis of ADP heptose reveals β-configuration as active form

To firmly establish the identity of the 618 peak as the NF-κB–stimulating factor, we decided to synthesize β-ADP heptose both chemically and enzymatically. For chemical synthesis, we applied 2 strategies: β-H1P was either coupled directly to AMP morpholidate (18) or to activated AMP by *in situ* generation of 2-imidazolyl-1,3-dimethylimidazolinium chloride, which converts the 5′phosphate ester of AMP to the reactive phosphorimidazolide (19). Both methods worked well, yielding moderate amounts of the expected β-ADP heptoses (l and d isomers), which eluted simultaneously from reversed-phase UPLC as compared with the active compound from *H. pylori* extracts (unpublished results).

**Figure 3:**
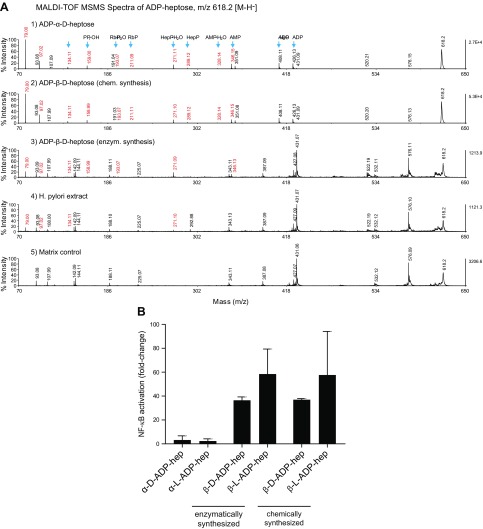
NF-κB–stimulating compound in *H. pylori* lysates is β-ADP heptose. *A*) MALDI-TOF MSMS spectra of ADP heptoses, *m/z* 618.2 [M-H]; corresponding fragment ions are labeled *1*) Reference spectrum: α-d-ADP heptose; *2*) β-d-ADP heptose from H1P or AMP morpholidate synthesis; *3*) β-d-ADP heptose from H1P/HldE enzymatic synthesis; *4*) β-d-ADP heptose from purified *H. pylori* extracts; *5*) Matrix control. *B*) AGS NF-κB luciferase reporter cells were stimulated with chemically synthesized ADP-α-d-heptose and β-d-ADP heptose, and d- and l-*glycero* isomers as well as with enzymatically generated β-d-ADP heptose in d and l forms in the presence of a transfection reagent for 3 h at concentrations of 10 µM. Induction is shown as fold change compared with untreated control. Data are means ± sem of at least 2 independent replicates.

**Figure 4:**
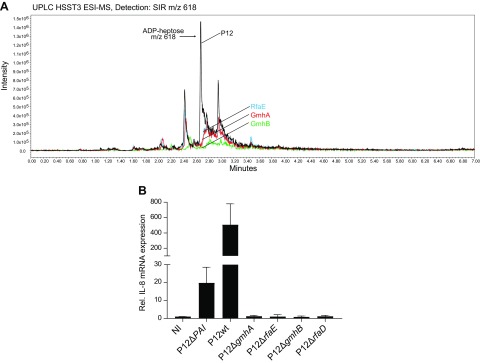
GmhB is essential for β-ADP heptose synthesis in *H. pylori* and necessary for NF-κB activation. *A*) Solid phase extracts from lysates of *H. pylori* WT and isogenic mutants Δ*gmhA*, Δ*gmhB*, and Δ*rfaE* were run over a Waters HSS T3 reversed-phase UPLC. Shown are the chromatograms in SIR mode for *m/z* 618 (negative mode). *B*) AGS cells were infected with *H. pylori* WT and indicated mutants at multiplicity of infection 100 for 3 h and analyzed for IL-8 induction by qRT-PCR. Data show relative induction compared with respective uninfected control. NI, not infected; Rel. relative. Data represent means ± sem of 2 independent replicates.

The *m/z* 618 [M-H]^−^ precursor ion was further analyzed by MALDI-TOF MSMS using chemically synthesized α-d-ADP heptose as reference (17). The same fragment ions could be observed in the *m/z* 618 precursor ion [M-H]^−^ as in the chemically synthesized ADP-α-d-heptose (Figs. 1–3*A*, see also Supplemental Fig. S4 for higher resolution spectra). Main fragment ions could be attributed to adenine (*m/z* 134), ribose-phosphate (*m/z* 211), heptose-phosphate (*m/*z** 289), AMP (*m/z* 346), and ADP (*m/z* 426). MALDI-TOF MSMS of the UPLC-fractionated SPE extract from *H. pylori* led to weak intensities of product ions (Figs. 3*A* and 4), strongly indicating that ADP heptose was indeed present in the stimulating fraction at a concentration up to 1 µM. Alkaline phosphatase treatment of these ADP heptoses, which were generated from the unprotected sugar phosphate, led to small amounts (1–5%) of a product with a mass of *m/z* 538 resulting from the loss of the heptose-phosphate group (unpublished results). We presume that coupling of activated AMP to the unprotected sugar led to small amounts of an adenylylated heptose-1-phosphate product, which became esterified at the 5′-phosphate of AMP at one of the hydroxyl groups of the heptose.

In order to rule out the possibility that the presence of an HBP analog (*e.g.*, 7′-adenylyl-heptose-1-phosphate) could be responsible for the activating effect, we also synthesized ADP heptose starting from the *O*-acetyl protected sugar (l-*glycero*-d-*manno*-heptose-2,3,4,6,7-*O*-penta-acetate), which was phosphitylated using a phosphoramidite (see Materials and Methods) and coupled by the AMP morpholidate method as described by Zamyatina *et al.* (17). Coupling of the *O*-acetyl protected sugar 1-phosphate to AMP morpholidate afforded acetylated ADP heptose (both anomeric forms), which was carefully deprotected, affording β-ADP heptose in low yields. As an alternative approach, we generated β-ADP heptose chemoenzymatically by either phosphorylating d-H7P or d- or l-β-H1P with purified recombinant HldE and GmhB from *H. pylori* in the presence of ATP (Fig. 3*A*, panel 3). The enzymatic conversion of the heptose monophosphates proceeded nearly quantitatively to ADP heptose.

To provide firm evidence that ADP heptose is the immunostimulatory molecule present in *H. pylori* lysates, epithelial cells were treated with different ADP heptoses. Both chemoenzymatically and chemically synthesized β-ADP heptoses (d- and l-*glycero* isomers) were able to stimulate NF-κB, whereas α-ADP heptose, even at higher concentrations, was not (Fig. 3*B* and unpublished results). Thus, in subsequent experiments, β-d-ADP heptose was used.

### ADP heptose is present in various gram-negative bacteria, and synthesis is dependent on GmhB in *H. pylori*

As heptose residues in the inner core of LPS and the ADP heptose synthesis pathway are conserved in most gram-negative bacteria, we tested lysates of different bacteria for the presence of ADP heptose. Of all the bacteria analyzed, ADP heptose was only detected in the gram-negative species (*Pseudomonas aeruginosa*, *Y. enterocolitica*, and *Salmonella typhimurium*) but not in the gram-positive ones (*Streptococcus pneumoniae* and *Listeria monocytogenes*) (Supplemental Fig. S5*A*). This finding confirms the role of ADP heptose as a general immunostimulatory molecule present in several pathogenic bacteria.

Interestingly, it has been reported before that a mutant in the *gmhB* gene coding for the GmhB that dephosphorylates HBP in the ADP heptose synthesis pathway is still capable of inducing an immune response (25). We therefore created an *H. pylori* mutant deficient of GmhB. We analyzed this mutant together with other mutants of the ADP heptose synthesis pathway (GmhA and RfaE) for the presence of ADP heptose by LC-MS. This compound could only be detected in WT bacteria but not in the GmhA, GmhB, and RfaE mutants (**Fig. 4*A***). When tested for their capacity to induce IL-8 in AGS cells, only WT bacteria were able to induce NF-κB (Fig. 4*B*), despite the fact that all mutants showed T4SS activity as indicated by cytoxin-associated gene A translocation after infection (Supplemental Fig. S5*B*).

### Proinflammatory activity of ADP heptose

**Figure 5:**
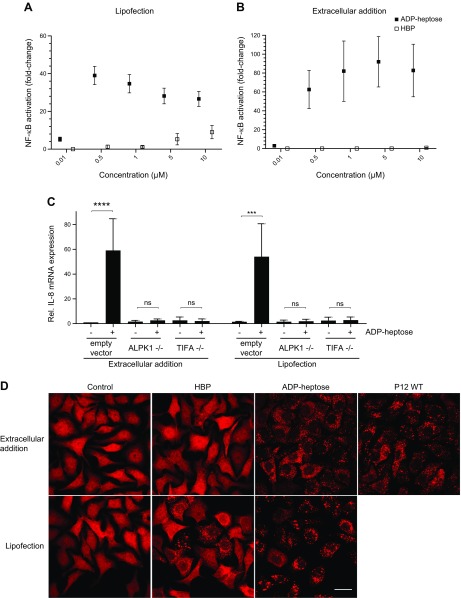
β-ADP heptose activates the ALPK1-TIFA axis at nanomolar concentrations. *A*, *B*) Treatment of AGS NF-κB luciferase reporter cells with β-d-ADP heptose and HBP in presence (*A*) or absence (*B*) of a transfection reagent for 3 h at indicated concentrations. Induction is shown as fold-change compared with an untreated control. Data represent means ± sem of 3 independent experiments. *C*) ALPK1^−/−^ and TIFA^−/−^ cells were stimulated with β-d-ADP heptose at a concentration of 10 µM in presence or absence of transfection reagent for 3 h or left untreated and analyzed for IL-8 induction by qRT-PCR. Data represent means ± sem of at least 2 independent replicates. *D*) AGS cells stably overexpressing tdTomato-TIFA were left untreated (control), treated with β-d-ADP heptose or HBP in presence or absence of a transfection reagent or infected with P12 WT at MOI 100 for 3 h. Formation of TIFAsomes was analyzed by confocal microscopy. Ns, not significant; NT, untreated. Scale bar, 20 µm. ***P ≤ 0.001, ****P ≤ 0.0001, Student’s *t* test.

Previous studies have shown that HBP needs to be delivered to the host cell cytoplasm for recognition and subsequent NF-κB activation (8, 9, 26). To our surprise, when we incubated an AGS reporter cell line with β-ADP heptose in the absence of transfection reagent, we observed a robust NF-κB response, whereas HBP only stimulated the cells when delivered into the cytoplasm by a transfection reagent (**Fig. 5*A, B***). Additionally, we observed that β-ADP heptose activates NF-κB at concentrations as low as 10–50 nM, which is around 100-fold lower than the concentration required for HBP (Fig. 5*A, B*). This corroborates the notion that ADP heptose, which is present in bacteria at sufficiently high concentrations, rather than HBP, constitutes the NF-κB–inducing factor.

To compare the activation level between lipofected and extracellularly added ADP heptose, we analyzed induction of IL-8 and did not observe significant differences (Supplemental Fig. S6*A*). Additionally, we challenged the cells with equimolar amounts of *E. coli* LPS. We did not observe activation of NF-κB, neither when added extracellularly nor when transfected into the cell, underlining the specific role of ADP heptose in inducing inflammation in epithelial cells (Supplemental Fig. S6*A*).

We hypothesized that β-ADP heptose might share the same signaling pathway as HBP, activating NF-κB *via* the ALPK1-TIFA axis. We therefore treated AGS WT as well as AGS TIFA^−/−^ and ALPK1^−/−^ cells with β-ADP heptose and measured the induction of IL-8. We observed that NF-κB activation triggered by β-ADP heptose was completely abrogated in ALPK1 and TIFA knockout cells (Fig. 5*C*). Additionally, we wanted to assess whether ADP heptose used different signaling routes after extracellular challenge and intracellular delivery. In both conditions, ALPK1 and TIFA knockout cells failed to show induction of IL-8, which is indicative of a common mechanism of activation (Fig. 5*C*).

To further demonstrate an activation of the ALPK1-TIFA axis by β-ADP heptose, we incubated a stably tdTomato-TIFA–overexpressing cell line with β-ADP heptose. Induction of TIFAsomes could be observed in *H. pylori* WT–infected cells, cells treated by addition of or lipofection with β-ADP heptose, and in cells lipofected with HBP (Fig. 5*D*). Quantifying the activation of cells confirmed our finding in that an induction of TIFAsomes is much less pronounced in cells transfected with HBP than in cells challenged with ADP heptose or infected with *H. pylori* (Supplemental Fig. S6*B*). We therefore conclude that β-ADP heptose does indeed activate the ALPK1-triggered TIFA phosphorylation to cause NF-κB activation and that β-ADP heptose rather than HBP is the main proinflammatory PAMP in *H. pylori*. It is very likely that β-ADP heptose also plays a decisive role in other infections.

## DISCUSSION

The identification of immune stimulatory molecules and downstream pathways is crucial for understanding how cells can sense infection and initiate an immune response. Here, we reveal β-ADP heptose as the key proinflammatory effector molecule of highly pathogenic, T4SS-positive *H. pylori*. We show that β-ADP heptose is present in *H. pylori* at more than 10-times higher concentrations than HBP. At the same time, it is far more potent, activating ALPK1-TIFA–controlled innate immune responses in epithelial cells at 100-fold lower concentrations. Its capacity to stimulate NF-κB was confirmed with chemically and enzymatically synthesized β-ADP heptoses (d- and l-*glycero* isomers). Recognition is specific, as evidenced by the fact that the α-configurated forms elicited no response. By directly demonstrating that ADP heptose is present in gram-negative bacteria at sufficient concentrations, these results complement the recent demonstration by Zhou *et al.* (12) that ADP heptose is the ligand for ALPK1.

Former studies focusing on HBP, including ours, showed that permeabilization of the membrane by lipofection or electroporation is necessary for a robust HBP-triggered NF-κB response, underlining the role of HBP in invasive infections (8, 9, 25, 26). Interestingly, with β-ADP heptose, activation occurred to the same extent even when added extracellularly without permeabilization of the cell membrane. Nevertheless, activation by *H. pylori* itself was still dependent on its functional cag pathogenicity island type-4 secretion system. We note, however, that infection with *H. pylori *cag PAI deletion mutants did induce very minor levels of NF-κB activation, suggesting that although the ADP heptose cannot be translocated, small amounts may indeed be released (*i.e.*, if some bacteria are lysed during *in vitro* experiments). This is reminiscent of *N. gonorrheae*, in which the release of respective PAMPs may be substantially facilitated by bacterial autolysis (8).

Interestingly, regardless of whether NF-κB activation occurred *via* HBP upon membrane permeabilization or ADP heptose upon extracellular addition, both routes were dependent on ALPK1 and TIFA in target cells. The exact mechanism of uptake or translocation of extracellular β-ADP heptose into host cells now requires further investigation. A dynamin-dependent uptake has been proposed for HBP stimulation of Jurkat cells (8), and a similar mechanism could apply in the case of ADP heptose. The study by Zhou *et al.* (12) also suggested that HBP is converted inside the host cell to heptose-7-phosphate, which is also able to activate ALPK1. In support of this theory, we too observed NF-κB activation in cells treated with ADP heptose-7-phosphate (unpublished results). It seems unlikely, however, that variations in the mode of ALPK1-TIFA axis activation by the 2 molecules would lead to major changes in the regulation of target genes.

The generation of ADP heptose from d-sedoheptulose-7-phosphate involves a multienzyme cascade (Supplemental Fig. S1*A*). Previous studies have shown that deletion of *gmhA* and *rfaE* abolish the induction of NF-κB (9, 25, 26). Yet, a mutant in *gmhB*, the phosphatase that converts HBP into heptose-1-phosphate, a precursor for β-ADP heptose synthesis, still showed NF-κB activation (25, 26). In our experiments, we could not detect ADP heptose in lysates of *H. pylori* strains deficient of *gmhA*, *rfaE*, and *gmhB.* Accordingly, we did not observe activation of NF-κB by these strains, underlining the predominant role of ADP heptose for rapid *H. pylori–*induced NF-κB activation. We cannot exclude that, depending on the *Helicobacter* strain, some *gmhb* mutants are still capable of ADP heptose synthesis *via* an alternative pathway, explaining the preserved NF-κB activation reported elsewhere (25, 26). Such a compensatory mechanism is known from *E. coli*, in which the *gmhB* gene function can at least partially be compensated by other phosphatases (7).

We were also able to demonstrate the presence of ADP heptose in lysates of other gram-negative bacteria, confirming its role as a common immunostimulatory molecule. Although β-ADP heptose serves as a central building block of the inner core biosynthesis of LPS in gram-negative bacteria, it has also been detected in some gram-positive bacteria (7, 27). Interestingly, our comparative experiments showed that, in epithelial cells, ADP heptose is far more potent than LPS for activating NF-κB. It is also tempting to speculate that, besides NF-κB activation, the activation of other downstream targets may differ depending on the involvement of the upstream pathways. Stimulation of NF-κB activation by common inducers, such as IL-1β or TNF-α, involves canonical pathways, whereas stimulation by ADP heptose and HBP leads to the formation of the large TIFAsome complex, which, as we revealed previously (9), associates multiple signaling molecules that may have a capacity to stimulate additional routes, such as MAPK pathways. Likewise, a recent report showed that signaling *via* TIFA can lead to activation of the inflammasome (28). It will certainly be interesting to explore the spectrum of ALPK1-TIFA–dependent pathways in greater depth. The relevance of β-ADP heptose as a PAMP *in vivo*, its potential usefulness as an adjuvant, and its importance in other clinical settings constitute further questions of relevance.

## Supplementary Material

This article includes supplemental data. Please visit *http://www.fasebj.org* to obtain this information.

## Abbreviations

β-H1P: β-l-*glycero*-d-*manno*-heptose-1-phosphate
ADP heptose: ADP-l-*glycero*-β-d-*manno*-heptose
AEC: 3-amino-9-ethylcarbazole
ALPK1: α-kinase 1
CIP: calf intestine alkaline phosphatase
ESI: electrospray ionization
fw: forward
GmhA: Sedoheptulose-7-phosphate isomerase
GmhB: d-*glycero*-β-d-*manno*-heptose-1,7-bisphosphate-7-phosphatase; H7P, d-*glycero*-d-*manno*-heptose-7-phosphate
HBP: heptose-1,7-bisphosphate
LB: Luria-Bertani
LC: liquid chromatography
MALDI: matrix-assisted laser desorption/ionization
MS: mass spectrometry
MSMS: tandem MS
OD_550/600_: optical density at 550/600 nm
PAMP: pathogen-associated molecular pattern
PDE: phosphodiesterase
qRT-PCR: quantitative RT-PCR
rv: reverse
SIR: single ion recording
SPE: solid phase extraction
T4SS: type-IV secretion system
TEAB: triethylammonium bicarbonate
TFA: trifluoroacetic acid
TIFA: TRAF-interacting protein with forkhead-associated domain
TOF: time of flight
UPLC: ultraperformance liquid chromatography
WT: wild type

## References

[1] Janeway C. A. Jr (1989) Approaching the asymptote? Evolution and revolution in immunology. Cold Spring Harb. Symp. Quant. Biol. 54, 1–13 10.1101/sqb.1989.054.01.003 2700931

[2] Medzhitov R. (2007) Recognition of microorganisms and activation of the immune response. Nature 449, 819–826 17943118 10.1038/nature06246

[3] Baccala R., Gonzalez-Quintial R., Lawson B. R., Stern M. E., Kono D. H., Beutler B., Theofilopoulos A. N. (2009) Sensors of the innate immune system: their mode of action. Nat. Rev. Rheumatol. 5, 448–456 19597511 10.1038/nrrheum.2009.136

[4] DiDonato J. A., Mercurio F., Karin M. (2012) NF-κB and the link between inflammation and cancer. Immunol. Rev. 246, 379–400 22435567 10.1111/j.1600-065X.2012.01099.x

[5] Newton K., Dixit V. M. (2012) Signaling in innate immunity and inflammation. Cold Spring Harb. Perspect. Biol. 4, a006049 22296764 10.1101/cshperspect.a006049 PMC3282411

[6] Jost P. J., Ruland J. (2007) Aberrant NF-kappaB signaling in lymphoma: mechanisms, consequences, and therapeutic implications. Blood 109, 2700–2707 17119127 10.1182/blood-2006-07-025809

[7] Kneidinger B., Marolda C., Graninger M., Zamyatina A., McArthur F., Kosma P., Valvano M. A., Messner P. (2002) Biosynthesis pathway of ADP-L-glycero-beta-D-manno-heptose in Escherichia coli. J. Bacteriol. 184, 363–369 11751812 10.1128/JB.184.2.363-369.2002 PMC139585

[8] Gaudet R. G., Sintsova A., Buckwalter C. M., Leung N., Cochrane A., Li J., Cox A. D., Moffat J., Gray-Owen S. D. (2015) Innate immunity. Cytosolic detection of the bacterial metabolite HBP activates TIFA-dependent innate immunity. Science 348, 1251–1255 26068852 10.1126/science.aaa4921

[9] Zimmermann S., Pfannkuch L., Al-Zeer M. A., Bartfeld S., Koch M., Liu J., Rechner C., Soerensen M., Sokolova O., Zamyatina A., Kosma P., Mäurer A. P., Glowinski F., Pleissner K. P., Schmid M., Brinkmann V., Karlas A., Naumann M., Rother M., Machuy N., Meyer T. F. (2017) ALPK1- and TIFA-dependent innate immune response triggered by the Helicobacter pylori type IV secretion system. Cell Rep. 20, 2384–2395 28877472 10.1016/j.celrep.2017.08.039

[10] Milivojevic M., Dangeard A. S., Kasper C. A., Tschon T., Emmenlauer M., Pique C., Schnupf P., Guignot J., Arrieumerlou C. (2017) ALPK1 controls TIFA/TRAF6-dependent innate immunity against heptose-1,7-bisphosphate of gram-negative bacteria. PLoS Pathog. 13, e1006224 28222186 10.1371/journal.ppat.1006224 PMC5336308

[11] Pfannkuch L., Hurwitz R., Traulsen J., Kosma P., Schmid M., Meyer T. (2018) ADP heptose, a novel pathogen associated molecular pattern associated with Helicobacter pylori type 4 secretion. bioRxiv. 10.1101/405951 PMC6662969 31075211

[12] Zhou P., She Y., Dong N., Li P., He H., Borio A., Wu Q., Lu S., Ding X., Cao Y., Xu Y., Gao W., Dong M., Ding J., Wang D. C., Zamyatina A., Shao F. (2018) Alpha-kinase 1 is a cytosolic innate immune receptor for bacterial ADP-heptose. Nature 561, 122–126 30111836 10.1038/s41586-018-0433-3

[13] Backert S., Ziska E., Brinkmann V., Zimny-Arndt U., Fauconnier A., Jungblut P. R., Naumann M., Meyer T. F. (2000) Translocation of the Helicobacter pylori CagA protein in gastric epithelial cells by a type IV secretion apparatus. Cell. Microbiol. 2, 155–164 11207572 10.1046/j.1462-5822.2000.00043.x

[14] Livak K. J., Schmittgen T. D. (2001) Analysis of relative gene expression data using real-time quantitative PCR and the 2(-Delta Delta C(T)) method. Methods 25, 402–408 11846609 10.1006/meth.2001.1262

[15] Güzlek H., Graziani A., Kosma P. (2005) A short synthesis of D-glycero-D-manno-heptose 7-phosphate. Carbohydr. Res. 340, 2808–2811 16263101 10.1016/j.carres.2005.10.003

[16] Borio A., Hofinger A., Kosma P., Zamyatina A. (2017) Chemical synthesis of the innate immune modulator – bacterial d - glycero -β- d - manno- heptose-1,7-bisphosphate (HBP). Tetrahedron Lett. 58, 2826–2829

[17] Zamyatina A., Gronow S., Puchberger M., Graziani A., Hofinger A., Kosma P. (2003) Efficient chemical synthesis of both anomers of ADP L-glycero- and D-glycero-D-manno-heptopyranose. Carbohydr. Res. 338, 2571–2589 14670718 10.1016/s0008-6215(03)00319-7

[18] Wagner G. K., Pesnot T., Field R. A. (2009) A survey of chemical methods for sugar-nucleotide synthesis. Nat. Prod. Rep. 26, 1172–1194 19693414 10.1039/b909621n

[19] Tanaka H., Yoshimura Y., Jørgensen M. R., Cuesta-Seijo J. A., Hindsgaul O. (2012) A simple synthesis of sugar nucleoside diphosphates by chemical coupling in water. Angew. Chem. Int. Ed. Engl. 51, 11531–11534 23065967 10.1002/anie.201205433

[20] Nagata S., Hamasaki T., Uetake K., Masuda H., Takagaki K., Oka N., Wada T., Ohgi T., Yano J. (2010) Synthesis and biological activity of artificial mRNA prepared with novel phosphorylating reagents. Nucleic Acids Res. 38, 7845–7857 20660478 10.1093/nar/gkq638 PMC2995060

[21] Sekine T., Kawashima E., Ishido Y. (1995) Synthesis of [5′-11C]ribonucleoside and -2′-deoyribonucleoside derivatives from D-ribose. Nucleic Acids Symp. Ser. 34, 11–12 8841527

[22] Sambrook J. R., Russell D. W. (2001) Molecular Cloning: A Laboratory Manual, Cold Spring Harbor Laboratory Press, New York

[23] Belogolova E., Bauer B., Pompaiah M., Asakura H., Brinkman V., Ertl C., Bartfeld S., Nechitaylo T. Y., Haas R., Machuy N., Salama N., Churin Y., Meyer T. F. (2013) Helicobacter pylori outer membrane protein HopQ identified as a novel T4SS-associated virulence factor. Cell. Microbiol. 15, 1896–1912 23782461 10.1111/cmi.12158 PMC3797234

[24] Han J., Tschernutter V., Yang J., Eckle T., Borchers C. H. (2013) Analysis of selected sugars and sugar phosphates in mouse heart tissue by reductive amination and liquid chromatography-electrospray ionization mass spectrometry. Anal. Chem. 85, 5965–5973 23682691 10.1021/ac400769g PMC3989532

[25] Stein S. C., Faber E., Bats S. H., Murillo T., Speidel Y., Coombs N., Josenhans C. (2017) Helicobacter pylori modulates host cell responses by CagT4SS-dependent translocation of an intermediate metabolite of LPS inner core heptose biosynthesis. PLoS Pathog. 13, e1006514 28715499 10.1371/journal.ppat.1006514 PMC5531669

[26] Gall A., Gaudet R. G., Gray-Owen S. D., Salama N. R. (2017) TIFA signaling in gastric epithelial cells initiates the cag type 4 secretion system-dependent innate immune response to Helicobacter pylori infection. MBio 8, e01168-17 28811347 10.1128/mBio.01168-17 PMC5559637

[27] Tang W., Guo Z., Cao Z., Wang M., Li P., Meng X., Zhao X., Xie Z., Wang W., Zhou A., Lou C., Chen Y. (2018) d-Sedoheptulose-7-phosphate is a common precursor for the heptoses of septacidin and hygromycin B. Proc. Natl. Acad. Sci. USA 115, 2818–2823 29483275 10.1073/pnas.1711665115 PMC5856511

[28] Lin T. Y., Wei T. W., Li S., Wang S. C., He M., Martin M., Zhang J., Shentu T. P., Xiao H., Kang J., Wang K. C., Chen Z., Chien S., Tsai M. D., Shyy J. Y. (2016) TIFA as a crucial mediator for NLRP3 inflammasome. Proc. Natl. Acad. Sci. USA 113, 15078–15083 27965388 10.1073/pnas.1618773114 PMC5206521

