# Supplementary material for: ADP heptose, a novel pathogen-associated molecular pattern identified in Helicobacter pylori
Source: FASEB J. 2019 Jun 21;33(8):9087–99. doi: 10.1096/fj.201802555R (PMC6662969; doi:10.1096/fj.201802555R)
Supplement: Supplementary file 1 [file fj.201802555R.sd1.pdf]

Supplementary Information

Supplementary Figure 1: Schematic overview of the ADP-heptose synthesis pathway and the identification of ADP-heptose in *H. pylori* lysates

(A) Schematic overview of ADP-L-glycero-β-D-manno-heptose synthesis starting from D-sedoheptulose 7-P. (B-E) Schematic overview of identification of ADP-heptose in *H. pylori* lysates. (B) After acid hydrolysis of HPB only the O-glyosidically bound phosphate is cleaved. After AEC devitalization, products were analyzed by mass spectrometry and a mass of 485 $[M+H]^+$ was detected. After CIP treatment, both phosphate residues are cleaved, resulting in the detection of a mass of 405 $[M+H]^+$ after AEC derivatization. (C) *H. pylori* lysates were treated with TFA for acidic hydrolysis and derivatized with AEC, leading to the detection of the specific 485 $[M+H]^+$ mass indicative for the presence of HBP, yet at low concentrations. Additionally a mass of 405 $[M+H]^+$ was detected, indicative of a yet unidentified sugar. (D*) H. pylori* lysates were separated by UPLC and screened for NF-κB activation capacity. The activating fraction was further analyzed. After acid hydrolysis a sugar derivate with a mass of 405 $[M+H]^+$ could be detected that was resistant to CIP cleavage indicative of a sugar other than HBP. (E) ADP-heptose after acid hydrolysis and AEC derivatization shows a mass of 405 $[M+H]^+$ and is resistant to CIP cleavage.

Supplementary Figure 2: MALDI-TOF MSMS reference spectra of D-glycerol-β-D-manno-heptose-AEC adducts.

(A) and (B) UPLC fraction of chemically synthesized β-D-HBP that was derivatized with AEC after dephosphorylation with TFA or CIP and analyzed by MALDI-TOF MSMS as described in Figure 1. (A) m/z 405 selected, (B) m/z 485 selected.

Supplementary Figure 3: MALDI-TOF MSMS-spectra of *H. pylori* extract (carbon graphite eluate) after UPLC purification and CIP and PDE digestion of *H. pylori* extracts.

The NF-κB stimulating fraction (No. 35) was dephosphorylated by TFA or CIP and derivatized with AEC. (A) MALDI-TOF MSMS spectra of D-glycerol-β-D-manno-heptose-AEC adduct, m/z 405 selected), (B) m/z 485 selected. (C) *H. pylori* extract (SPE) was left untreated (black

line, 1), treated with calf intestine alkaline phosphatase (CIP, blue line, 2), or with *Crotalus adamanteus* phosphodiesterase (PDE, green line, 3). Digests were run over a Waters HSS T3 reversed phase UPLC. Shown are the chromatograms in SIR (Single Ion Recording) mode for m/z 618 (negative mode).

Supplementary Figure 4: MALDI-TOF MSMS Spectra of ADP-heptoses m/z 618.2 [M-H]

Higher resolution of spectra shown in Figure 3A; (A) Upper panel (1): Reference spectrum: α-D-ADP-heptose, blue arrows: main fragment ions, middle panel (2): ADP-β-D-heptose from H1P/AMP-morpholidate synthesis, lower panel (5): matrix control.

Supplementary Figure 5: ADP-heptose is present in lysates of other Gram-negative bacteria

(A) Solid phase extracts from lysates of *Pseudomonas aeruginosa*, *Yersinia enterocolitica, Salmonella typhimurium, Streptococcus pneumonia* and *Listeria monocytogenes* were run over a Waters HSS T3 reversed phase UPLC. Shown are the chromatograms in SIR (Single Ion Recording) mode for m/z 618 (negative mode). (B) Starved AGS cells were infected with *H. pylori* P12 WT or indicated isogenic mutants for 3 h. Cells were analyzed for translocation of CagA as evidenced by the appearance of phospho-CagA. Actin served as loading control. One representative blot of two replicates is shown.

Supplementary Figure 6: ADP-heptose but not LPS activates epithelial cells after extracellular challenge or lipofection

A) AGS cells were stimulated with ADP-β-D-heptose, β-D-HBP or LPS (Ultrapure LPS, E. coli 0111:B4, Invivogen) at indicated concentrations in the presence or absence of transfection reagent for 3 h and analyzed for IL-8 induction by RT-qPCR. Data shows relative induction compared to respective transfected or untreated control. Data represent mean ± SEM of two independent replicates, statistical analysis of induction to respective control; ns: not significant, Student's t-test. (B) Quantification of cells showing TIFAsomes after treatment with *H. pylori*

P12, or ADP-β-D-heptose or β- HBP in presence or absence of transfection reagent for 3 h. Data represents mean ± SEM of two independent replicates.

Supplementary Table 1: Mass peaks of NF-κB stimulating fraction from *H. pylori* after mild acidic hydrolysis, or CIP-treatment followed by AEC derivatization

HBP (D-glycero-β-D-manno-heptose 1,7-bisphosphate) and purified fractions (UPLC, Fr.35) from *H. pylori* extracts were derivatized with 3-amino-9-ethylcarbazole (AEC) after being hydrolyzed with trifluoroacetic acid (TFA) or alkaline calf intestine phosphatase (CIP). AEC-heptose derivatives were separated by reversed phase LC and analyzed by MALDI-TOF MSMS spectrometry (positive mode). Shown are the peak intensities of the most prominent fragment ion m/z 223 of the precursor ions m/z 405 (Supplementary Figures 2A and 3A) and m/z 485 (Supplementary Figures 2B and 3B), respectively.

Supplementary Figure 1

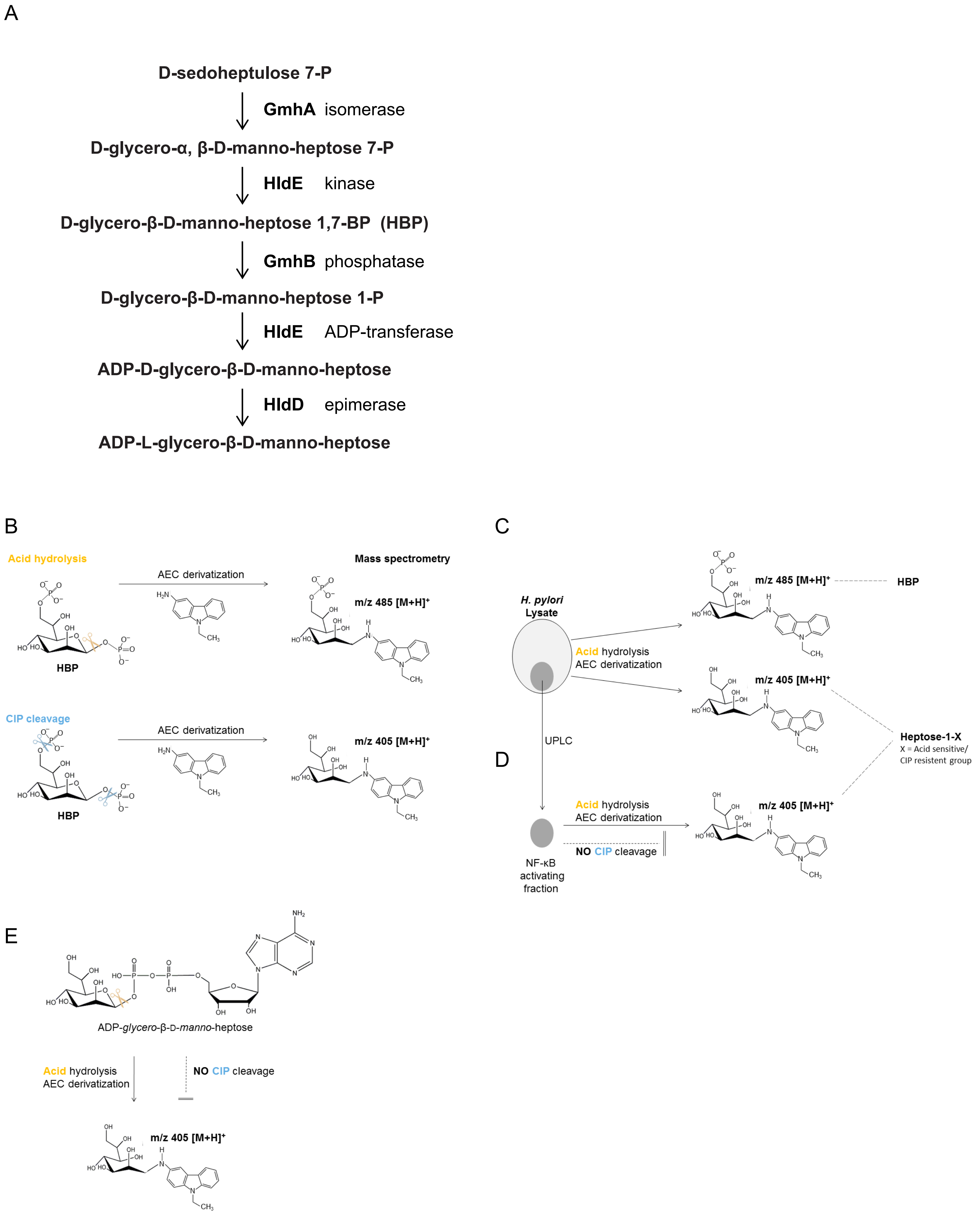

Supplementary Figure 2

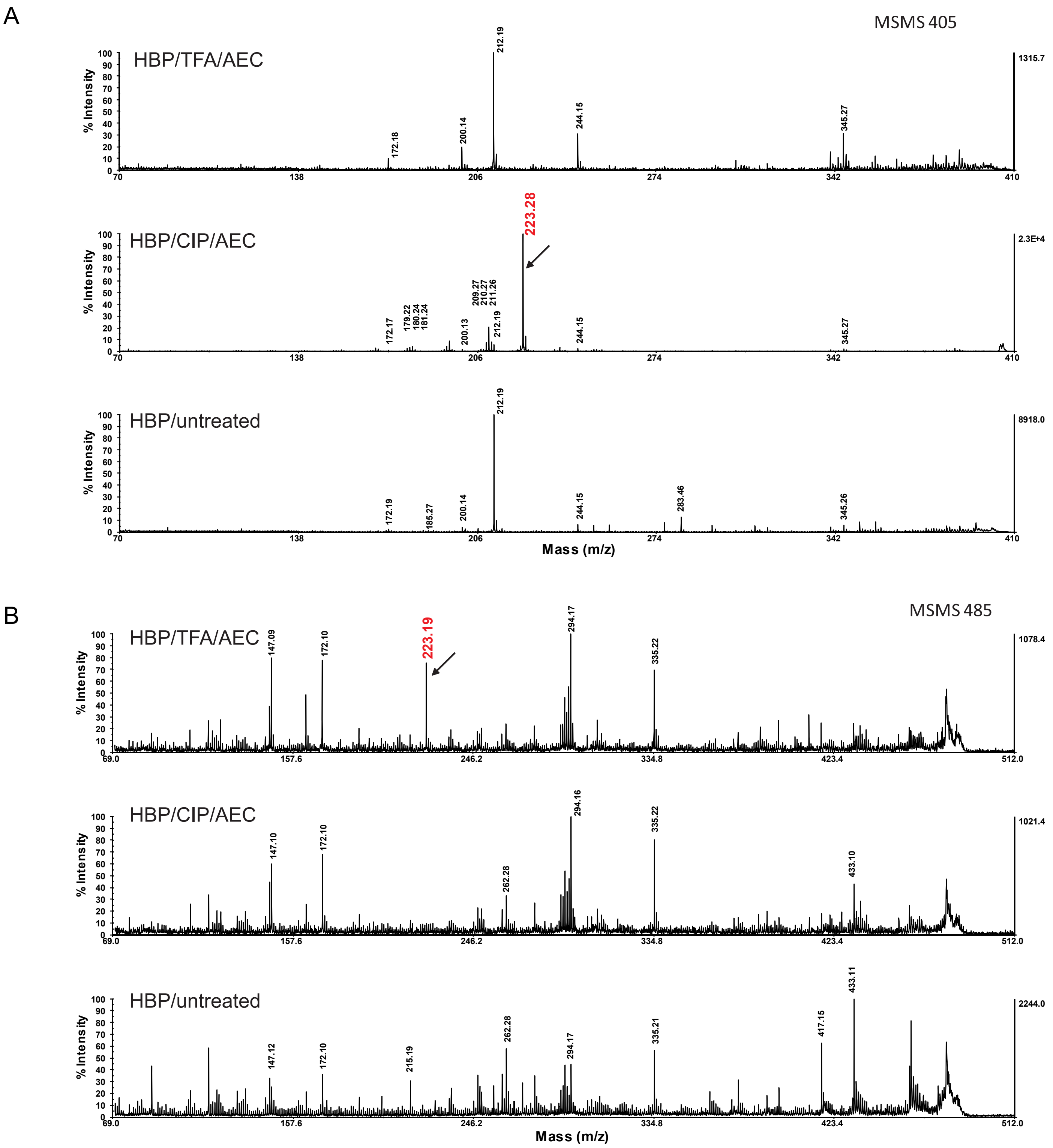

Supplementary Figure 3

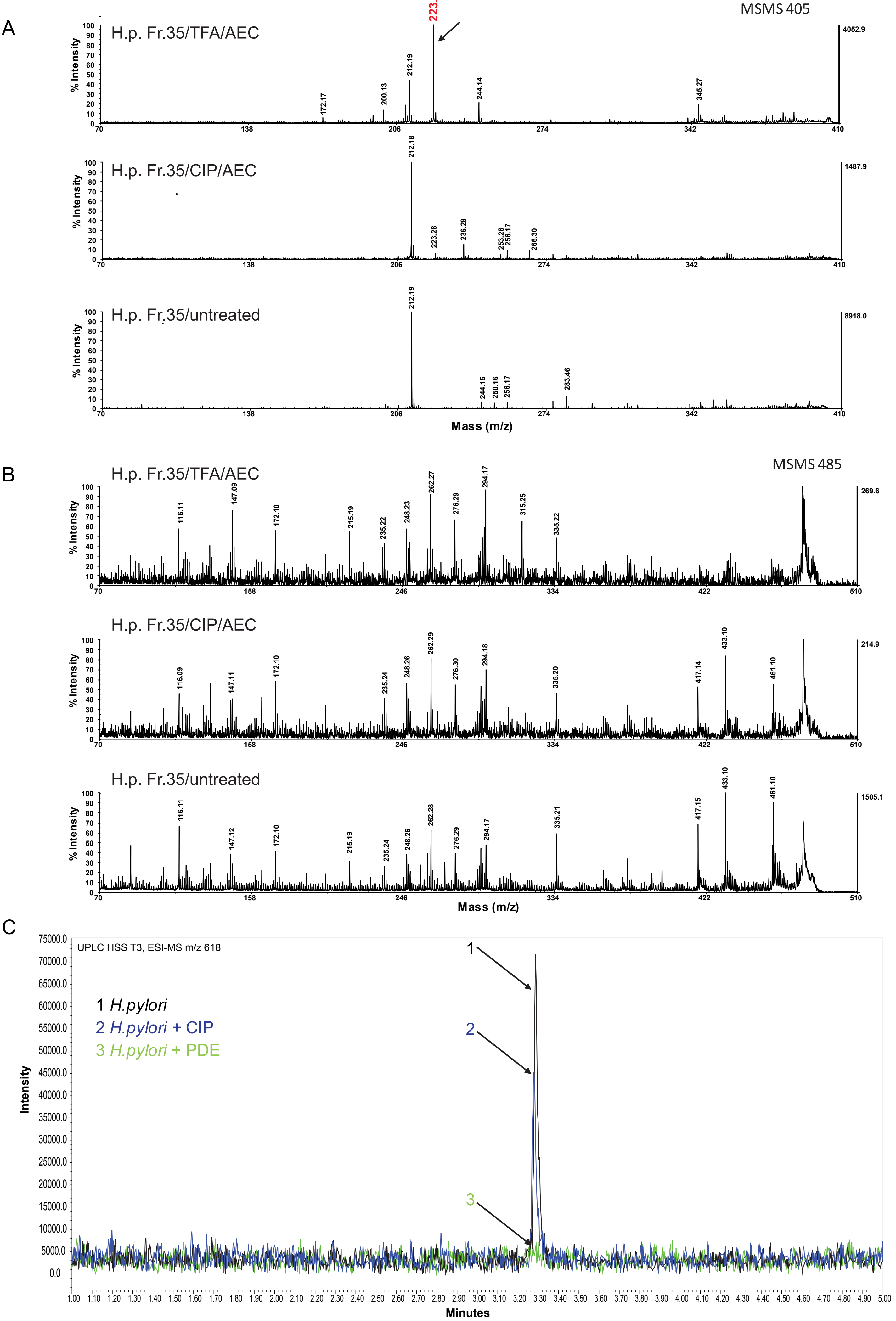

Supplementary Figure 4

A

MALDI-TOF MSMS Spectra of ADP-heptose, m/z 618.2 [M-H$^-$]

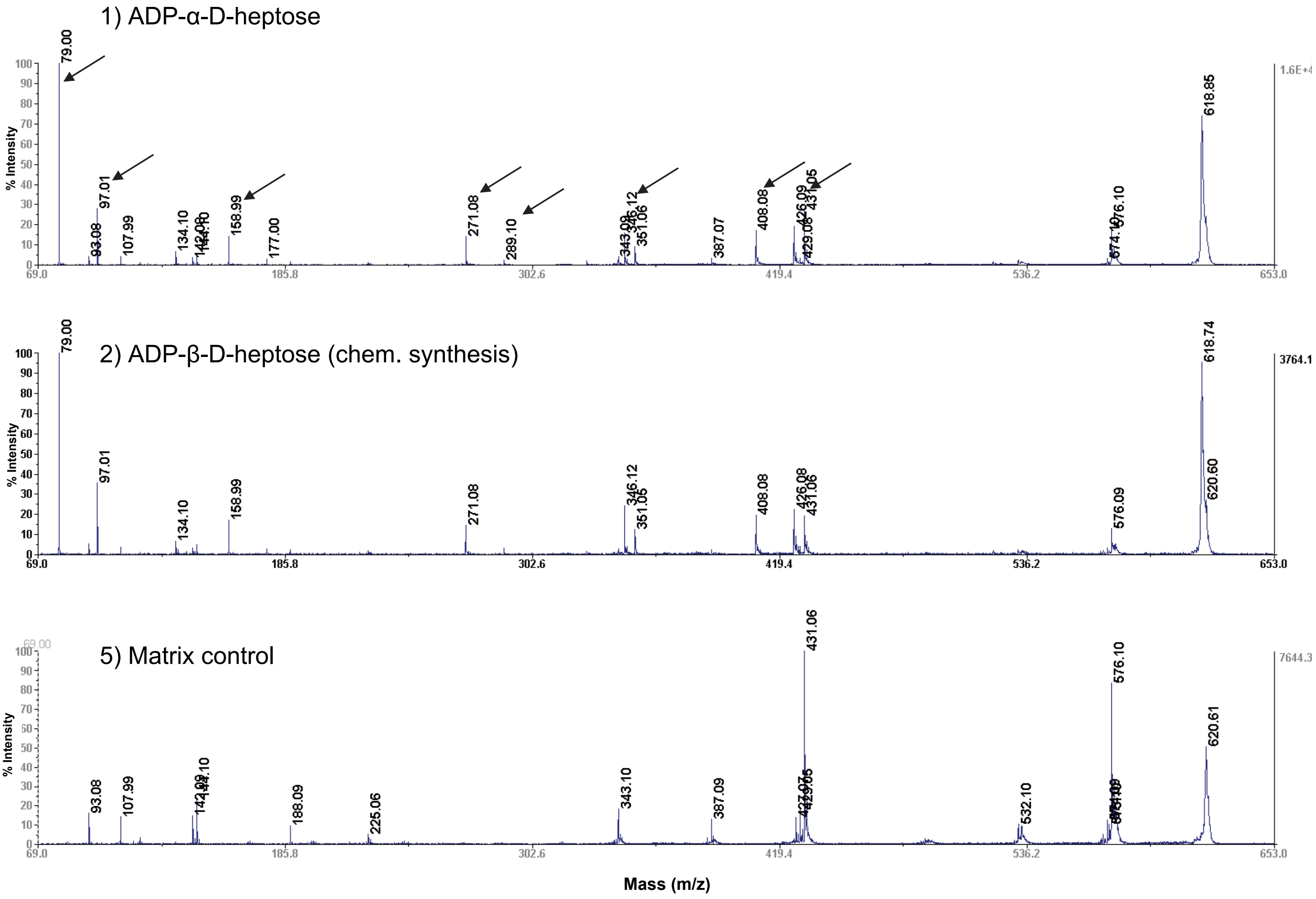

Supplementary Figure 5

A

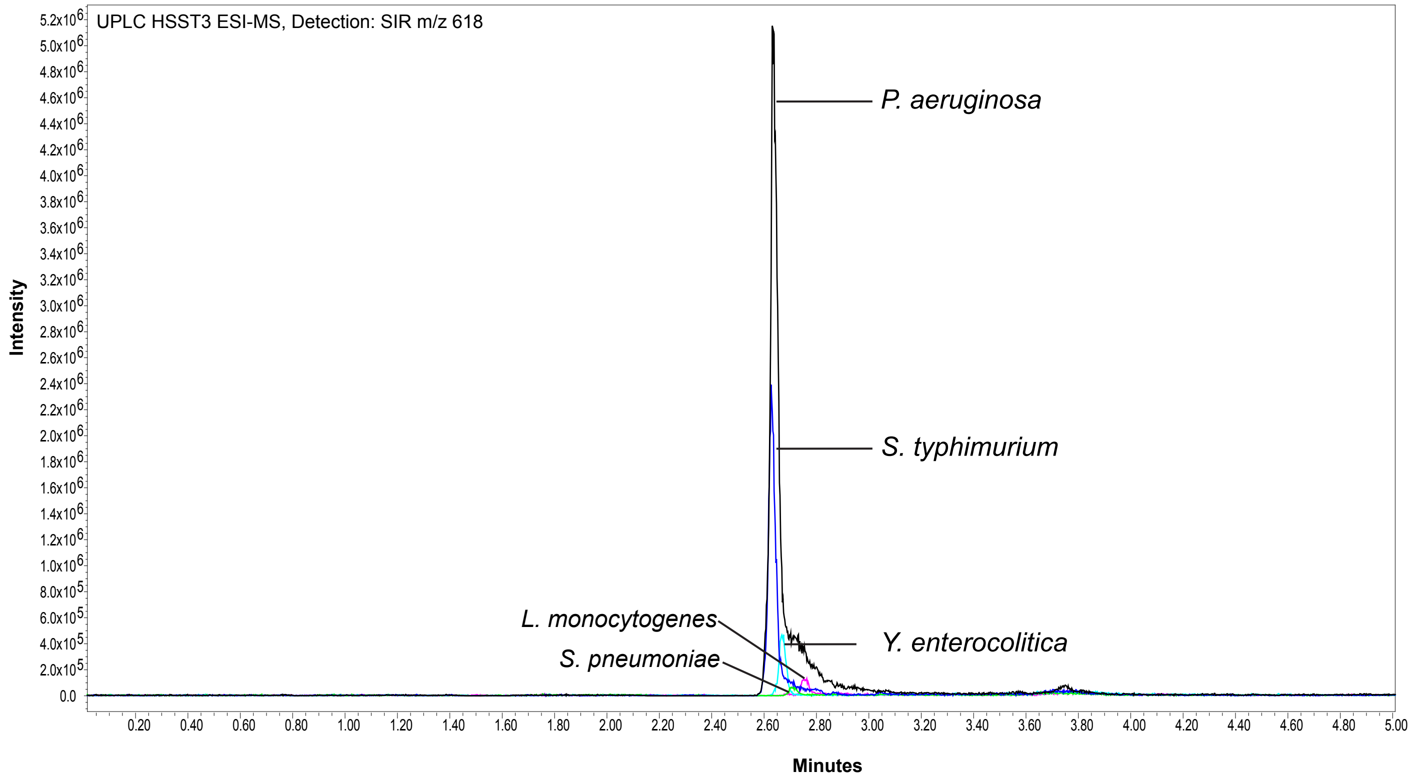

B

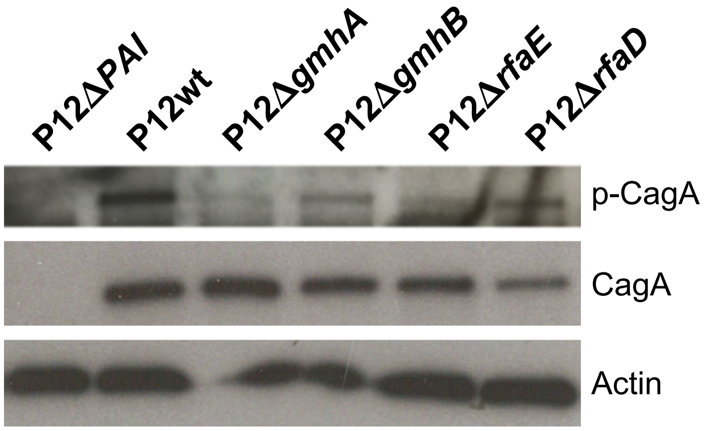

Supplementary Figure 6

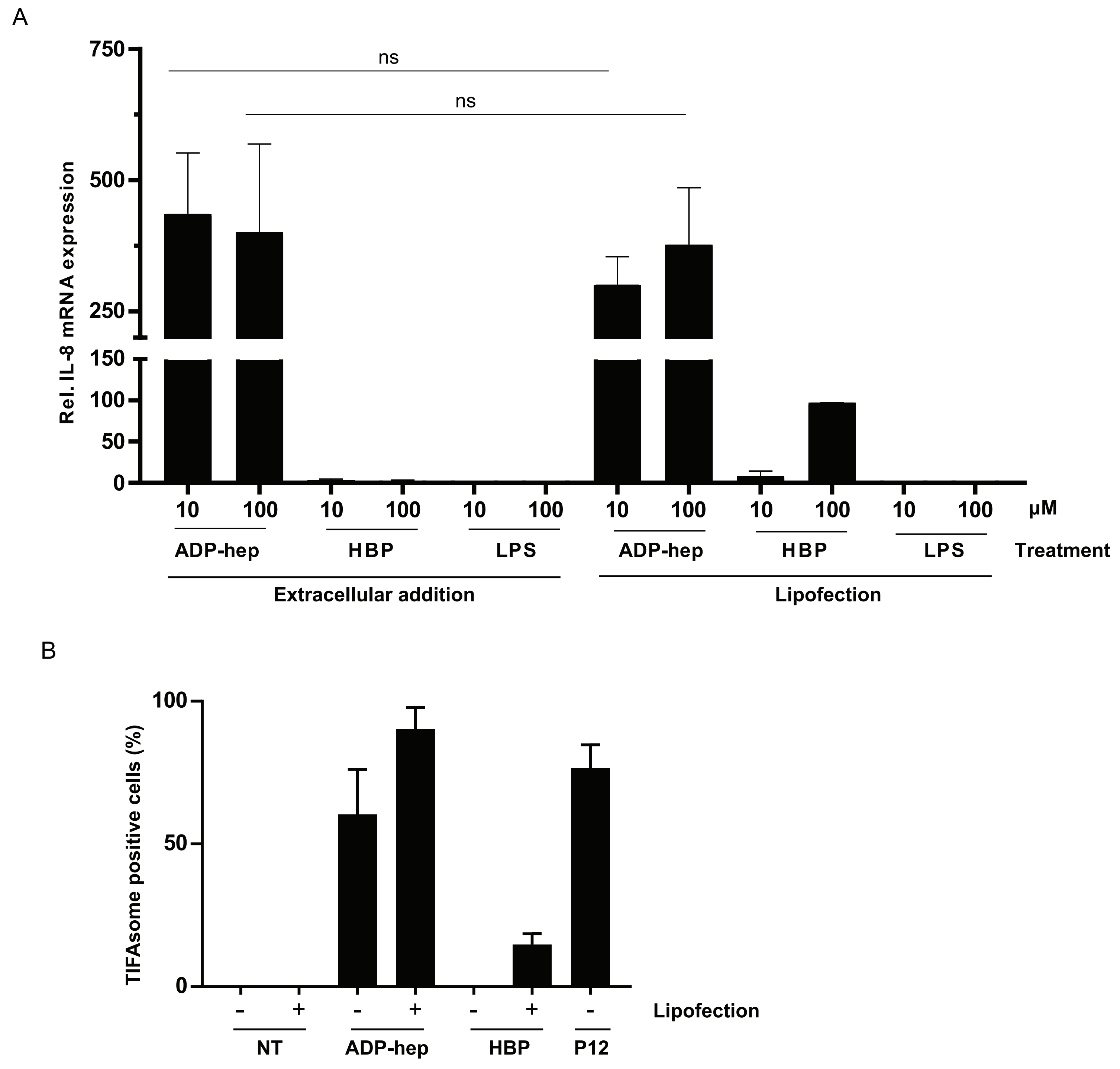

Table S1

	Precursor ion m/z $[M+H]^+$	Fragment ion m/z $[M+H]^+$	Precursor ion m/z $[M+H]^+$	Fragment ion m/z $[M+H]^+$
	**405.1**	**223.1**	**485.1**	**223.1**
HBP/TFA	0	0	75	783
HBP/CIP	12.850	30.959	0	88
HBP/untreated	0	0	0	0
H.p extract; Fr.35 /TFA	**1.230**	**5.665**	38	88
H.p. extract; Fr.35/CIP	9	137	0	0
H.p. extract/untreated	0	0	0	0
